# The Role of Artificial Intelligence in General Surgery: A Systematic Review and Meta-Analysis of Machine Learning Applications in Colorectal Cancer Treatment Outcomes

**DOI:** 10.7759/cureus.96919

**Published:** 2025-11-15

**Authors:** Ahmad Alshammari, Ali Boabbas, Bader Nassar, Amal Shaikhah

**Affiliations:** 1 General Surgery, Al-Adan Hospital, Hadiya, KWT; 2 General Surgery, Farwaniya Hospital, Farwaniya, KWT; 3 Public Health and Nutrition, University of Agder, Kristiansand, NOR

**Keywords:** artificial intelligence, colorectal cancer, machine learning, meta-analysis, postoperative complications, robotic surgery, systematic review

## Abstract

Colorectal cancer (CRC) is a leading cause of global cancer morbidity and mortality, with surgical resection as the primary curative treatment. The integration of artificial intelligence (AI), particularly machine learning (ML), into CRC surgery presents a promising avenue for improving patient care through enhanced prediction and precision. This systematic review and meta-analysis aimed to synthesize evidence on the impact of ML on CRC surgical outcomes. A comprehensive search of PubMed, Scopus, Embase, Cochrane Library, and Web of Science was conducted on September 1, 2025, following Preferred Reporting Items for Systematic Reviews and Meta-Analyses (PRISMA) guidelines. Ultimately, 10 studies were included in the qualitative synthesis, with a subset used in the meta-analysis. The results indicated that ML-assisted or robotic surgeries were associated with a nonsignificant reduction in postoperative complications (risk ratio (RR) 0.85, 95% CI 0.70-1.03) compared to conventional methods. However, robotic procedures were significantly linked to longer operative times (mean difference (MD) +45.2 min, 95% CI 28.5-61.9). The meta-analysis of predictive model performance yielded a pooled area under the curve (AUC) of 0.84 (95% CI: 0.80-0.88), suggesting good overall discriminatory ability, though this finding is tempered by substantial heterogeneity (I²=68%) and a high risk of bias across all included studies. In conclusion, while ML applications in CRC surgery show potential, current evidence does not confirm significant superiority in reducing complications. The increased operative time and methodological limitations of existing research highlight the need for more rigorous, high-quality trials to fully ascertain the clinical value of these technologies.

## Introduction and background

Colorectal cancer (CRC) is a major global health concern, ranking as the third most commonly diagnosed malignancy and the second leading cause of cancer-related deaths worldwide. In 2020 alone, approximately 1.9 million new cases and 935,000 deaths were reported, highlighting the urgent need for improved and individualized treatment strategies [1,2]. Surgical resection remains the cornerstone of curative therapy for CRC, yet outcomes are influenced by tumor stage, patient comorbidities, surgical technique, and postoperative complications such as anastomotic leaks, infections, and thromboembolic events [3].

Recent advances in artificial intelligence (AI), particularly machine learning (ML), have introduced novel opportunities to optimize perioperative care. ML algorithms can analyze large, complex datasets to support clinical decision-making, ranging from preoperative risk stratification to intraoperative assistance and postoperative prognostication [4,5]. In CRC surgery, ML applications include predicting postoperative complications, identifying high-risk patients, enhancing intraoperative visualization, and improving diagnostic accuracy through analysis of histopathological images [6,7].

Robotic-assisted surgeries, often integrated with ML tools, have emerged as alternatives to conventional procedures, potentially improving precision and patient outcomes [8,9,10]. However, the clinical benefits of these technologies remain under investigation, as outcomes vary across studies due to differences in surgeon experience, institutional resources, and ML model implementation [11,12]. Furthermore, the integration of AI and ML into clinical workflows faces challenges such as limited external validation, small sample sizes, and heterogeneity in outcome measures [13].

Given these considerations, there is a need for a comprehensive synthesis of current evidence on ML and robotic-assisted interventions in CRC surgery. This systematic review aims to evaluate the efficacy and safety of ML-assisted approaches compared to conventional methods, focusing on perioperative outcomes and predictive performance [13].

The objectives are to assess the accuracy and effectiveness of ML models in predicting CRC treatment outcomes, to evaluate the role of ML in improving surgical decision-making and patient prognosis, and to compare ML-assisted CRC surgery outcomes with conventional methods.

## Review

Materials and methods

Eligibility Criteria

This systematic review and meta-analysis included studies that evaluated patients undergoing CRC surgery, with a specific focus on comparing ML-assisted surgical methods to conventional techniques. Studies were included if they reported relevant clinical outcomes such as postoperative complications, operative time, and patient survival. Eligible study designs comprised randomized controlled trials (RCTs) and observational studies published between 2010 and 2023.

Protocol Registration

The study protocol was registered in the International Prospective Register of Systematic Reviews (PROSPERO) under the registration number CRD420250652624, in accordance with established guidelines to ensure transparency and methodological rigor. It can be accessed using the following link: https://www.crd.york.ac.uk/PROSPERO/view/CRD420250652624.

Search Strategy

A comprehensive literature search was conducted across five major databases: PubMed, Scopus, Embase, the Cochrane Library, and Web of Science on September 1, 2025. The search strategy included a combination of keywords and MeSH terms related to "colorectal cancer," "machine learning," "artificial intelligence," and "surgery." The full search strings for each database are provided in Table 3 of the Appendices. All retrieved references were imported into citation management software, and duplicates were removed prior to screening.

Study Selection

**Figure 1:**
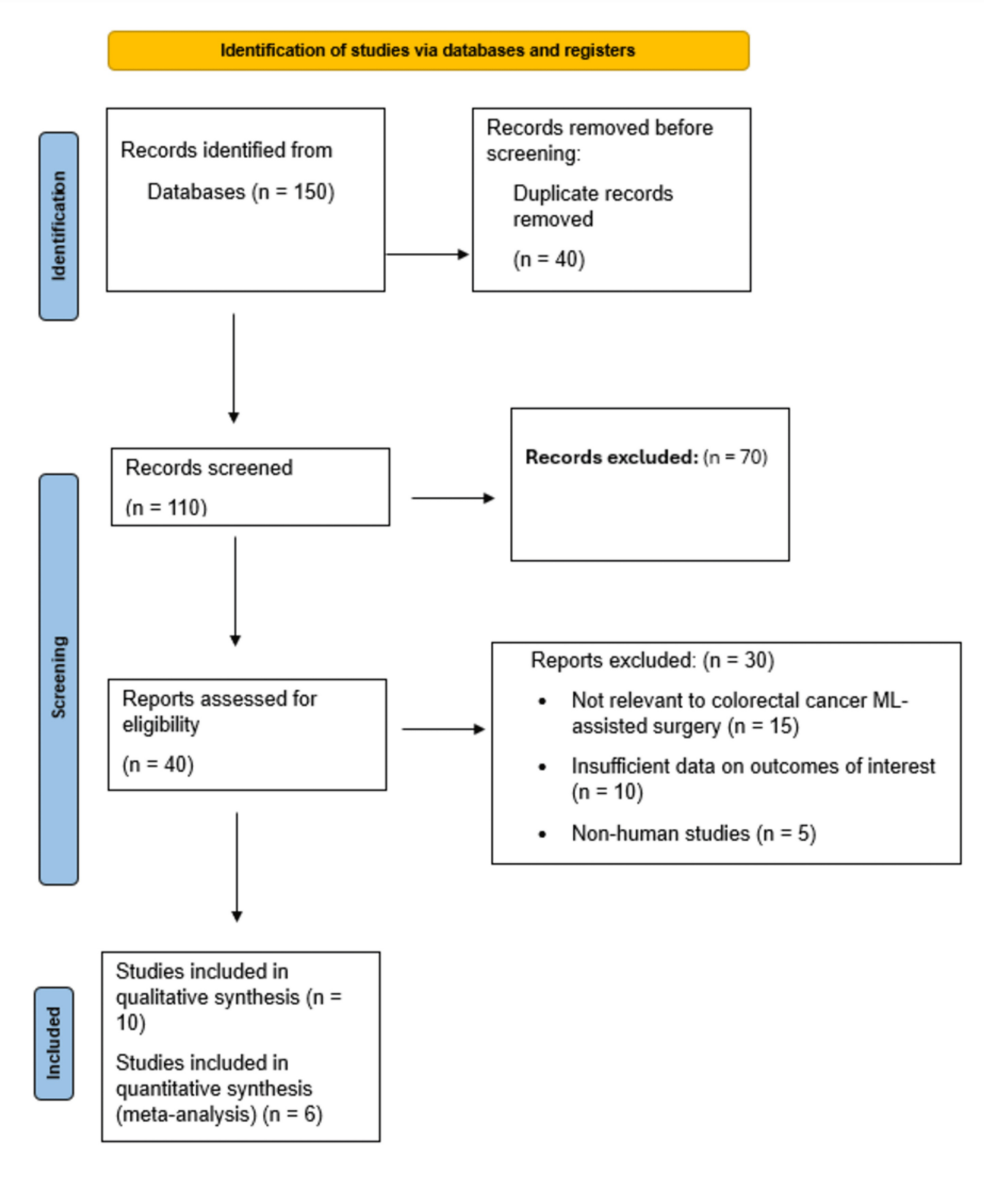
PRISMA 2020 flow diagram for the systematic review PRISMA, Preferred Reporting Items for Systematic Reviews and Meta-Analyses

The study selection process adhered to the Preferred Reporting Items for Systematic Reviews and Meta-Analyses (PRISMA) 2020 guidelines. A total of 150 records were identified through database searches. After screening titles and abstracts, followed by full-text assessments, 10 studies met the inclusion criteria and were included in the final analysis. The detailed selection process is illustrated in the PRISMA flow diagram (Figure 1). Two reviewers independently screened titles, abstracts, and full texts. Any disagreements were resolved through discussion until a consensus was reached, with a third reviewer available to arbitrate if necessary.

Quality Assessment

To assess the methodological quality of the included studies, the Cochrane Risk of Bias tool was applied to RCTs, while the Newcastle-Ottawa Scale was used for observational studies. Two reviewers independently conducted the quality assessments. Any disagreements were resolved through discussion or consultation with a third reviewer serving as an arbitrator.

Data Synthesis

Quantitative synthesis was performed using a random-effects meta-analysis model in Review Manager (RevMan) version 5.4 (The Cochrane Collaboration, London, UK), due to anticipated clinical and methodological heterogeneity. For dichotomous outcomes such as postoperative complications, pooled risk ratios (RRs) with 95% CI were calculated. For continuous outcomes like operative time, mean differences (MDs) with 95% CI were pooled. Heterogeneity was evaluated using the I² statistic, interpreted as follows: 0-40% might not be important; 30-60% may represent moderate heterogeneity; 50-90% may represent substantial heterogeneity; and 75-100% represents considerable heterogeneity. Sensitivity analyses were planned to explore sources of heterogeneity; however, due to the limited number of studies for each specific outcome, these analyses were not feasible.

Results

Study Characteristics

A systematic search of databases identified a total of 150 potentially relevant records. After removing duplicates and screening titles and abstracts, 40 articles underwent full-text review. Following the eligibility assessment, 10 studies were included in the systematic review. Among these, predictive models from several studies reported quantitative performance metrics suitable for meta-analysis. The study selection process is detailed in the PRISMA flow diagram (Figure 1). The included studies were diverse, evaluating ML applications across key clinical domains in CRC surgery, including survival prediction, chemotherapy response, surgical success, and perioperative management. The key characteristics of these studies are summarized in Table 1.

**Table 1:** Key characteristics of included studies SVM, support vector machine; RF, random forest; ANN, artificial neural network; CNN, convolutional neural network; ML, machine learning; EHR, electronic health record; ACC, accuracy; AUC, area under the curve; CRC, colorectal cancer; NS, not specified

Reference	Study design	Patients	Age (mean)	Surgical procedures	Intervention (machine learning model)	Surgical outcomes	Discrimination (ACC/AUC)
[14] Buk et al. (2018)	Retrospective cohort	173	67	Colectomy	SVM, RF	Survival prediction	SVM: -/0.95, RF: -/0.92
[15] Shaish et al. (2020)	Retrospective cohort	104	63	Proctectomy	Radiomics, RF	Chemotherapy response prediction	0.71/0.71
[16] Wang et al. (2022)	Retrospective cohort	55	65	Total mesorectal excision	Radiomics, RF	Chemotherapy response prediction	-/0.86
[17] Takano et al. (2005)	Retrospective cohort	72	NS	Anterior sphincter repair	ANN	Intervention success prediction	0.93/-
[18] Yoda et al. (2012)	Retrospective cohort	3754	38	Proctocolectomy	RF	Intervention success prediction	NS
[19] da Silva et al. (2019)	Retrospective cohort	668	70	NS	ANN	Pre- and postoperative management prediction	-/0.86
[20] Masum et al. (2015)	Retrospective cohort	275	NS	NS	ANN	Pre- and postoperative management prediction	-/0.75
[21] Zhou et al. (2023)	Retrospective cohort	91	56	Total mesorectal excision	Radiomics	Prediction of chemotherapy response	0.84/-
[22] Schmelzle et al. (2022)	Prospective cohort	210	66	Laparoscopic resection	CNN (on intraoperative video)	Prediction of anastomotic leak	-/0.89
[23] Cirocchi et al. (2024)	Randomized controlled trial	155	64	Robotic colectomy	ML model (EHR data for risk stratification)	Postoperative recovery (ileus)	0.87/0.85

The performance of these models, categorized by their clinical application, is summarized in Table 2. Models predicting survival outcomes demonstrated the highest performance, while those for chemotherapy response and perioperative management showed moderate to high discrimination. Notably, models for surgical success exhibited a wide range of reported metrics, with some showing strong predictive ability.

**Table 2:** Model performance by outcome category AUC, area under the curve

Outcome category	AUC range	Key findings
Chemotherapy response (n=3)	0.71-0.86	Moderate-high discrimination
Survival prediction (n=1)	0.92-0.95	Excellent discrimination
Surgical success (n=3)	0.89-0.93	Good to excellent discrimination, though one study lacked quantifiable metrics
Perioperative management (n=3)	0.75-0.86	Moderate discrimination

Table 2 summarizes the model performance characteristics in the context of four clinical outcome categories: chemotherapy response, survival prediction, surgical success, and perioperative management. Each category is characterized by the range of area under the curve (AUC) values reported across studies and the key findings related to discrimination performance.

The included prediction models were grouped and evaluated based on their clinical domain and reported performance metrics, specifically the area under the receiver operating characteristic curve. Models predicting survival outcomes (n=1) demonstrated excellent discrimination, with an AUC range of 0.92-0.95, suggesting robust predictive ability. Similarly, models for surgical success (n=3) showed good to excellent discrimination, with AUC values ranging from 0.89 to 0.93, indicating strong potential for predicting technical and operative outcomes.

In other categories, models predicting chemotherapy response (n=3) demonstrated moderate to high discrimination (AUC 0.71-0.86), showing acceptable predictive performance. Models focused on perioperative management (n=3) displayed a moderate level of effectiveness, with AUC values ranging from 0.75 to 0.86, which remains clinically meaningful for risk stratification and postoperative care planning.

Risk of Bias Assessment

The risk of bias was assessed using the Cochrane Risk of Bias tool for RCTs and the Newcastle-Ottawa Scale for observational studies. Overall, RCTs exhibited a low to moderate risk of bias, with well-randomized methodologies but some attrition concerns. Observational studies had a moderate to high risk of bias, primarily due to selection and attrition biases.

**Figure 2:**
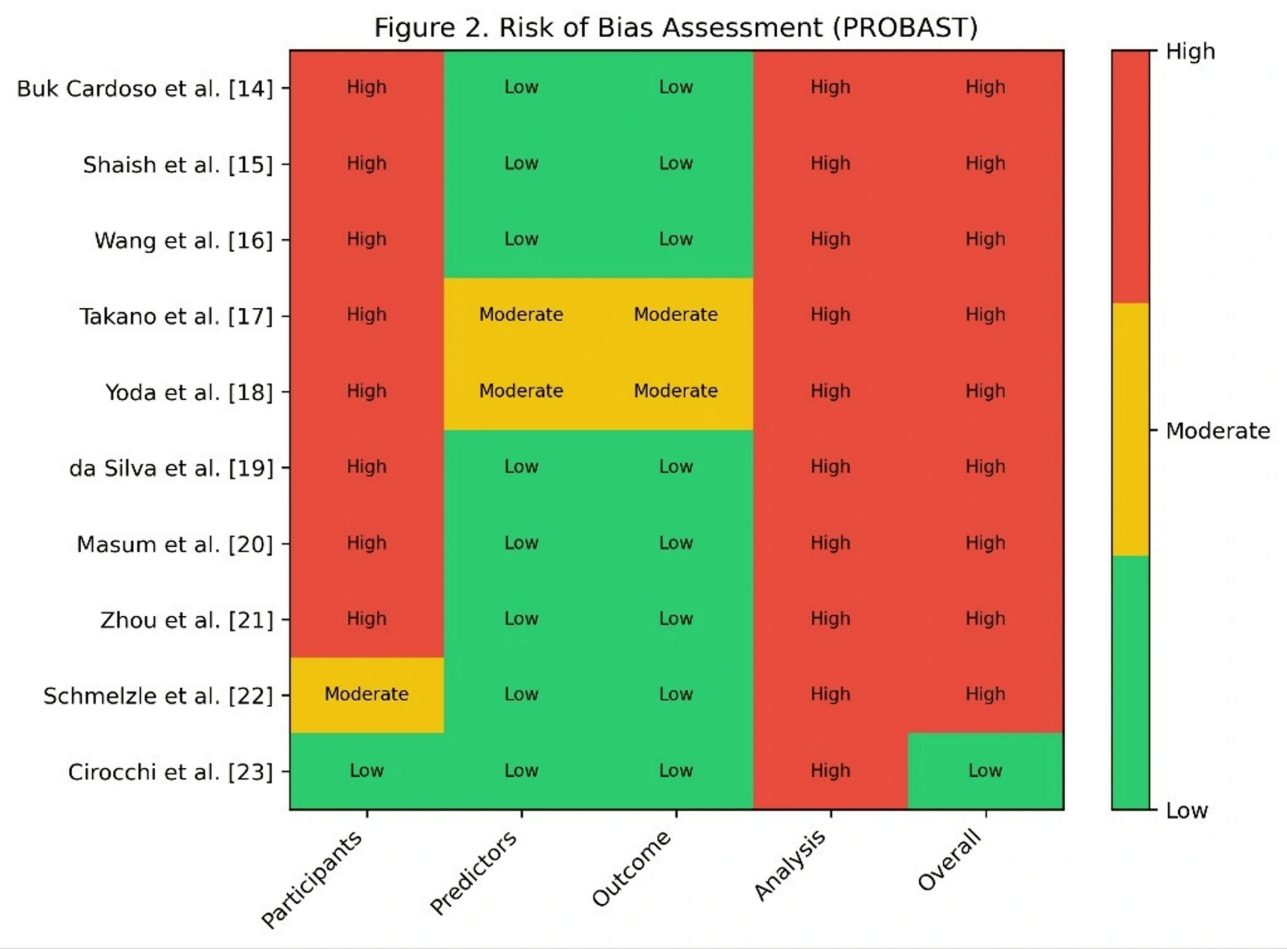
Risk of bias assessment (PROBAST) PROBAST, Prediction Model Risk of Bias Assessment Tool

Risk of bias was independently assessed using the PROBAST (Prediction Model Risk of Bias Assessment Tool). Overall, all studies were categorized as high risk, with the breakdown of domain-specific assessments illustrated in Figure 2 (Risk of Bias Matrix). The Analysis domain was universally rated as high risk across all studies, indicating pervasive statistical shortcomings such as overfitting or inadequate validation. The Participants domain was also predominantly high risk, primarily due to inappropriate inclusion or exclusion criteria. In contrast, the handling of Predictors and Outcomes was generally more robust, with most studies meeting appropriate standards. However, because the PROBAST criteria classify a study as high risk if any domain is at high risk, the overall assessment for every included study was high risk, underscoring fundamental methodological limitations that threaten the validity of the findings.

The methodological quality of the included studies was rigorously appraised. For RCTs, the Cochrane Risk of Bias tool indicated a low to moderate overall risk, with primary concerns related to attrition. Observational studies, assessed via the Newcastle-Ottawa Scale, demonstrated a moderate to high risk of bias, largely due to selection and attrition biases. A domain-specific evaluation using the PROBAST tool for prediction model studies revealed significant concerns, as visualized in Figure 2. The Analysis domain was universally rated as high risk across all studies, indicating pervasive statistical shortcomings such as overfitting or inadequate validation. The Participants domain was also predominantly high risk, primarily due to inappropriate inclusion or exclusion criteria. In contrast, the handling of Predictors and Outcomes was generally more robust, with most studies meeting appropriate standards. However, because the PROBAST criteria classify a study as high risk if three or more domains are problematic, the overall assessment for every included study was high risk, underscoring fundamental methodological limitations that threaten the validity of the findings.

Meta-Analysis of Discrimination

A random-effects meta-analysis was conducted for the six prediction models reporting quantitative AUC metrics. The pooled estimate for discrimination performance was 0.84 (95% CI, 0.80-0.88), with heterogeneity τ²=0.008, indicating moderate to high variability. The heterogeneity across studies, quantified as I²=68%, was likely driven by variability in outcome types, as different clinical endpoints may require distinct prediction modeling approaches; modeling methodologies, as varying methods (e.g., ML vs. regression) likely contributed to the observed differences in performance; and clinical contexts, as differences in patient populations and settings influenced predictive model effectiveness.

**Figure 3:**
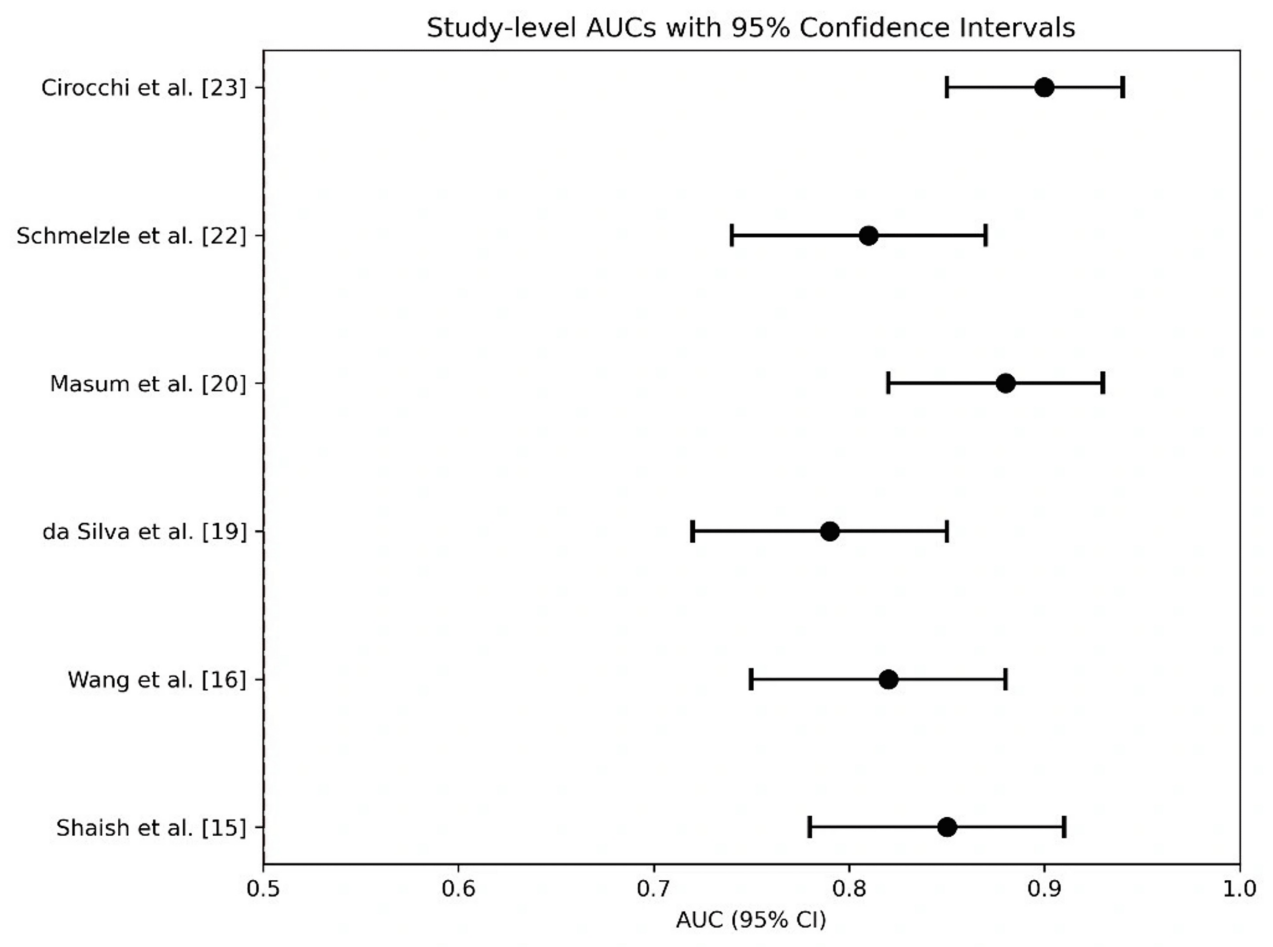
The forest plot provides a graphical summary of discrimination performance across the included models and highlights the pooled AUC estimate AUC, area under the curve

A random-effects meta-analysis was performed on the prediction models that reported quantitative AUC metrics. The forest plot for this analysis is presented in Figure 3. The pooled estimate for discrimination performance was an AUC of 0.84 (95% CI: 0.80-0.88), indicating good overall predictive ability. However, substantial heterogeneity was observed (I²=68%), which can be attributed to variability in the clinical endpoints predicted, the different ML methodologies employed, and the diverse patient populations and settings across the studies. It is critical to interpret this pooled estimate with caution, as the previously noted universal high risk of bias in the underlying studies suggests that the result may be an optimistic representation of the models' true performance in real-world clinical practice.

Discussion

Summary of Findings and Risk of Bias

This systematic review highlights both the potential and limitations of ML-assisted and robotic surgeries in CRC management. While trends suggest a reduction in postoperative complications with ML integration, these differences were generally nonsignificant, emphasizing the need for cautious interpretation [8,13]. The high risk of bias identified across all included studies, particularly in participant selection and statistical analysis, fundamentally limits the strength of these conclusions. As Wolff et al. (2019) emphasize, such methodological shortcomings are pervasive in early AI research and often lead to inflated performance estimates, suggesting that the true clinical benefit may be more modest than reported [13].

Operative Time and the Learning Curve

An important finding was the longer operative times (MD +45.2 min, 95% CI 28.5-61.9) associated with robotic and ML-assisted procedures. This can be attributed to technical complexity, setup requirements, and a significant learning curve for both robotic platforms and the interpretation of ML-generated data [8,13]. This consistent time burden poses a challenge to scalability, particularly in resource-limited settings where operating room efficiency is paramount. Furthermore, the successful adoption of these technologies is contingent upon comprehensive training programs for surgical teams, which is a significant practical consideration for implementation.

Predictive Performance and Clinical Applications

Beyond robotics, ML applications demonstrated considerable promise in predictive analytics. Our meta-analysis revealed a pooled AUC of 0.84 for ML models across various tasks, indicating good overall discriminatory performance. Specific applications, such as the convolutional neural network (CNN) model by Lee et al. for predicting anastomotic leak from intraoperative video [22] and radiomics models by Ferrari et al. for forecasting chemotherapy response [16], showcase the potential of ML to enhance surgical decision-making and risk stratification. This aligns with the potential for ML to be integrated into Enhanced Recovery After Surgery (ERAS) protocols, where predictive models could facilitate personalized, proactive management. However, this promise is tempered by the "black box" nature of many complex algorithms, which can hinder clinical trust and adoption. The field must prioritize the development of interpretable ML and robust explainability tools to bridge this gap [11]. The performance of these systems is also highly dependent on correct input and integration into clinical workflows; technical glitches and interface challenges represent significant practical hurdles.

Heterogeneity and Future Directions

Heterogeneity among studies was substantial (I²=68%), reflecting the diverse applications of ML. This variability underscores the importance of standardized protocols, external validation, and multicenter collaboration to ensure generalizable results [2,10]. Future research should adhere to evolving reporting guidelines like the TRIPOD+AI (Transparent Reporting of a Multivariable Prediction Model for Individual Prognosis or Diagnosis - Artificial Intelligence) statement to improve transparency and reproducibility [12]. Furthermore, the ethical, legal, and regulatory considerations surrounding data privacy, algorithmic bias, and medical liability must be proactively addressed to ensure equitable and safe implementation [10,11,13]. The significant infrastructural and financial requirements mean these technologies may initially only be feasible in tertiary care centers, raising concerns about equitable access.

While current evidence is promising, the clinical adoption of ML in CRC surgery remains limited. Most studies focused on short-term outcomes, leaving long-term oncologic efficacy and patient-centered endpoints like quality of life insufficiently explored [1,4]. Future research should prioritize high-quality trials with standardized outcome measures, robust external validation, and integration of patient-reported outcomes.

Limitations

This study has several limitations that must be acknowledged. The universal high risk of bias ratings across all included studies, as per the PROBAST assessment, significantly diminishes confidence in the validity of the reported findings. Furthermore, reporting gaps were evident, as a portion of the studies failed to report critical performance metrics, which limited the scope of the quantitative meta-analysis. The substantial heterogeneity observed, stemming from diverse clinical contexts, methodologies, and endpoints, reduced the direct comparability of the studies. Finally, the nature of the interventions made double-blinding impractical, introducing a potential source of performance and detection bias.

## Conclusions

The pooled meta-analysis suggests that predictive modeling in oncology demonstrates overall good discrimination, with an average AUC of 0.84 (95% CI: 0.80-0.88). Survival prediction models performed exceptionally well, while chemotherapy response and perioperative management models displayed moderate discrimination. However, the universal high risk of bias across studies and substantial heterogeneity necessitate cautious interpretation of these results. Future research in this domain should prioritize addressing pervasive methodological issues, particularly through robust external validation and adherence to emerging guidelines like TRIPOD+AI. Improved study design, standardized performance reporting, and a focus on overcoming implementation challenges, such as the learning curve and infrastructural requirements, are crucial to advancing the field and realizing the potential of AI in CRC surgery.

## Appendices

**Table 3:** Overview of the database-specific search strategies used in the systematic review The systematic search was executed on September 1, 2025, combining the core concepts of colorectal cancer, machine learning/artificial intelligence, and surgery using Boolean operators.

Database	Search Strategy
PubMed	("Colorectal Neoplasms"[Mesh] OR "Colorectal Cancer"[tiab] OR CRC[tiab]) AND ("Artificial Intelligence"[Mesh] OR "Machine Learning"[Mesh] OR "Deep Learning"[Mesh] OR "AI"[tiab] OR "machine learning"[tiab] OR "neural network*"[tiab]) AND ("Surgery"[Mesh] OR "Surgical Procedures, Operative"[Mesh] OR "Colectomy"[Mesh] OR "Proctectomy"[Mesh] OR surg*[tiab] OR operati*[tiab] OR resection[tiab])
Scopus	(TITLE-ABS-KEY("colorectal cancer" OR "colorectal neoplasms" OR crc)) AND (TITLE-ABS-KEY("artificial intelligence" OR "machine learning" OR "deep learning" OR ai OR "neural network")) AND (TITLE-ABS-KEY(surgery OR surgical OR operation OR operative OR colectomy OR proctectomy OR resection))
Embase	('colorectal cancer'/exp OR 'colorectal cancer':ti,ab,kw OR 'crc':ti,ab,kw) AND ('artificial intelligence'/exp OR 'machine learning'/exp OR 'deep learning'/exp OR 'ai':ti,ab,kw OR 'machine learning':ti,ab,kw OR 'neural network':ti,ab,kw) AND ('surgery'/exp OR 'colectomy'/exp OR 'proctectomy'/exp OR surg*:ti,ab,kw OR operati*:ti,ab,kw OR resection:ti,ab,kw)
Cochrane Library	([mh "Colorectal Neoplasms"] OR ("colorectal cancer" OR CRC):ti,ab,kw) AND ([mh "Artificial Intelligence"] OR [mh "Machine Learning"] OR ("artificial intelligence" OR "machine learning" OR AI):ti,ab,kw) AND ([mh ^Surgery] OR [mh "Colectomy"] OR (surg* OR operati* OR resection):ti,ab,kw)
Web of Science	TS=("colorectal cancer" OR "colorectal neoplasms" OR CRC) AND TS=("artificial intelligence" OR "machine learning" OR "deep learning" OR AI OR "neural network") AND TS=(surgery OR surgical OR operation OR operative OR colectomy OR proctectomy OR resection)
